# Clinical Phenotypes Associated to Engrailed 2 Gene Alterations in a Series of Neuropediatric Patients

**DOI:** 10.3389/fnana.2018.00061

**Published:** 2018-08-10

**Authors:** Francisco Carratala-Marco, Patricia Andreo-Lillo, Marta Martinez-Morga, Teresa Escamez-Martínez, Arancha Botella-López, Carlos Bueno, Salvador Martinez

**Affiliations:** ^1^Neuropaediatric Unit, University Hospital of San Juan de Alicante, Elche, Spain; ^2^Neuroscience Institute UMH-CSIC, CIBERSAM-ISCIII, Alicante, Spain; ^3^IMIB-Arrixaca, University of Murcia, Murcia, Spain

**Keywords:** *engrailed 2*, *EN2*, encephalic structural anomalies, genotype-genotype correlation, mental retardation, cerebral palsy, epilepsy, behavioral disorders

## Abstract

The engrailed homeobox protein (EN) plays an important role in the regionalization of the neural tube. EN distribution regulates the cerebellum and midbrain morphogenesis, as well as retinotectal synaptogenesis. In humans, the *EN1* and *EN2* genes code for the EN family of transcription factors. Genetic alterations in the expression of *EN2* have been related to different neurologic conditions and more particularly to autism spectrum disorders (ASD). We aimed to study and compare the phenotypes of three series of patients: (1) patients with encephalic structural anomalies (ESA) and abnormalities in the genomic (DNA) and/or transcriptomic (RNAm) of *EN2* (EN2-g), (2) ESA patients having other gene mutations (OG-g), and (3) ESA patients free of these mutations (NM-g).

**Subjects and Methods:** We have performed a descriptive study on 109 patients who suffer from mental retardation (MR), cerebral palsy (CP), epilepsy (EP), and behavioral disorders (BD), showing also ESA in their encephalic MRI. We studied genomic DNA and transcriptional analysis (cDNA) on *EN2* gene (EN2), and in other genes (OG): *LIS1, PTAFR, PAFAH1B2, PAFAH1B3, FGF8, PAX2, D17S379, D17S1866*, and *SMG6 (D17S5)*, as a routine genetic diagnosis in ESA patients.

**Results:** From 109 patients, fifteen meet the exclusion criteria. From the remaining 94 patients, 12 (12.8%) showed mutations in *EN2* (EN2-g), 20 showed mutations in other studied genes (OG-g), and 62 did not showed any mutation (NM-g). All EN2-g patients, suffered from MR, nine EP, seven BD and four CP. The proportions of these phenotypes in EN2-g did not differ from those in the OG-g, but it was significantly higher when comparing EN2-g with NM-g (MR: *p* = 0.013; EP: *p* = 0.001; BD: *p* = 0.0001; CP: *p* = 0.07, ns). Groups EN2-g and OG-g showed a 100 and a 70% of comorbidity, respectively, being significantly (*p* = 0.04) greater than NM-group (62.9%).

**Conclusion:** Our series reflects a significant effect of *EN2* gene alterations in neurodevelopmental abnormalities associated to ESA. Conversely, although these *EN2* related anomalies might represent a predisposition to develop brain diseases, our results did not support direct relationship between *EN2* mutations and specific clinical phenotypes.

## Introduction

The engrailed homeobox protein (en) plays an important role during development in the invertebrate segmentation, where it is required for the formation of posterior compartments in larva segments. In vertebrates, the EN protein has been involved the regionalization of the mid-hindbrain segment of the neural tube. Indeed, it is a reference transcription factor coding positional information in the cerebellum morphogenesis and retinotectal synaptogenesis (Martinez et al., 1991; Friedman and O’Leary, 1996; Cheng et al., 2010). Alexandra Joyner research group described the *En1* and *En2* homeobox-containing paralog genes in vertebrates and their expression during embryogenesis (Joyner et al., 1985; Joyner and Martinz, 1986). Then, Logan et al. (1989, 1992) mapped human *EN1* to chromosome 2 and EN2 to chromosome 7.

In the mouse, the *En2* expression in the mid-hindbrain segment is induced by Fgf8 signal from the isthmic organizer (Crossley et al., 1996; Martinez et al., 1999) and it is required to specify mesencephalic and cerebellar development, including serotoninergic and dopaminergic neurons in the raphe nuclei, substantia nigra, and ventral tegmental area (Millen et al., 1994; Wurst et al., 1994; Simon et al., 2005; Sonnier et al., 2007). In chick embryos, the expression gradient of *En2* in the tectum regulates the normal establishment of the retinotopic pattern of retinal innervation (Araki and Nakamura, 1999; Nakamura and Sugiyama, 2004).

The relationship between the autistic spectrum disorder (ASD) and *EN2* gene polymorphisms and mutations has been suggested since 1995 (Petit et al., 1995). More recently genome-wide association studies showed a potential role of *EN2* in ASD (Benayed et al., 2005, 2009). Moreover, the *EN2* abnormal expression and its methylation profile have been reported in the cerebellum of ASD patients (James et al., 2013, 2014; Choi et al., 2012, 2014; Jamuar et al., 2014). The mechanisms underplaying ASD related to *EN2* expression anomalies are still controversial (Yang et al., 2008, 2010). For instance, A-C specific polymorphism in *EN2* gene (A-C in *rs1861972* (A/G) and *rs1861973* (C/T)) has been associated to *EN2* over-expression in ASD individuals. Conversely, in Yang et al. (2010) A-C appears as a protective haplotype against ASD in Han Chinese ethnic group (see also Chien et al., 2011). The minor G-T haplotype is over-represented in unaffected siblings (Gharani et al., 2004; Benayed et al., 2005). The *EN2* overexpression may be a potential causal mechanism of developmental alterations in brain morphogenesis, generating a deregulated growth between the cerebellum and cortex (Rubenstein, 2010). This deregulation may represent an alteration in the spatial and temporal pattern of synaptogenesis that finally could determine behavioral phenotypes associated to ASD. Human brain postmortem studies and neuroimaging analysis have reported different degrees of cerebellar atrophy and cortical anomalies of dopaminergic/serotoninergic innervation in ASD brains (for review see, Palmen et al., 2004). Actually, Skefos et al. (2014) found that the mean overall Purkinje cell density was lower in the cases with autism as compared to controls. These structural alterations may be explained by abnormal development of the mid-hindbrain neural tube segment. In addition, brain anomalies due to the *EN2* haploinsufficiency have been also proposed as a cause of mental retardation in 7q terminal deletion syndrome (Frints et al., 1998; Linhares et al., 2013). Although these experimental and clinical data support the importance of the EN2 protein function in brain development, to date no studies have been published to try to establish an association between clinical and cerebral structural phenotypes and abnormalities in EN2 gene expression.

Therefore, we have studied the phenotypical characteristics of a series of patients with encephalic structural anomalies (ESA) and who showed abnormalities in the DNA and/or transcribed RNAm of *EN2* (EN2-g) and compare them with patients with: (1) ESA and other gene mutations (OG-g), including: *PAX2 and FGF8*, key factors for neural regionalization (Hidalgo-Sánchez et al., 2005), *LIS1* (also known as *PAFAH1B1*) and functionally related genes, involved in neuronal proliferation and migration in the developing brain (Spalice et al., 2009; Guerrini and Parrini, 2010); and (2) ESA and patients free of these type of mutations (NM-g), showing all of them similar clinical conditions.

## Materials and Methods

### Patients and Interventions

We have detected 12 patients with ESA and mutations in the *EN2* gene. These patients came from a group of 109 patients genetically studied because the presence of ESA in their cerebral hemispheres and/or brainstem in MRI tests. All the patients studied came from the population of 33291 children assigned to our Health Department Hospital of Health (17th Department) of the Valencian Universal Health Service of the autonomous Valencian Government. Patients were admitted to the outpatient neuropaediatric clinic of our secondary university hospital, in the period 2008–2014. The inclusion criteria to be admitted in this revision were: to suffer from MR, CP, EP, and BD, showing also structural abnormalities in their encephalic MRI studies. MR and BD were assessed by neuropediatricians and psychiatrists based on DSM-IV-R criteria, while CP and EP were diagnosed by neuropediatricians following CIE-10 and ILAE criteria. The MRI assessment was performed by the on duty neuroradiologist blind to the genotype condition of the patient. A third-party radiologist also reviewed some cases, with no variations between their diagnostic reports. From the 109 included patients, 15 patients who were diagnosed of ESA and the other inclusion criteria, but the clinical history reported suffering from some syndromic condition with known gene mutation or whose symptoms could be related to inflammatory or infectious diseases were removed from the analyzed series. Therefore, 94 patients have been revised for the present work.

A 2 mL blood part was removed from the complete blood sample taken from all the 109 patients as a routine laboratory workout in these kinds of patients, who need it as a compulsory element of the necessary diagnostic procedure, in patients referred to us with the indicated diagnostic suspicions.

The results of the analysis and the clinical data from patients were obtained with the informed consent of parents abiding the legislation that rules this type of studies in our institutions. At the time of blood extraction, we have obtained a written informed consent from the parents for the medical and genetic research and publication of the data. The reference code ethical approval to use human samples (including blood cells) is: 2016/VSC/PEA/00091(Responsible person of Ethical Committee: Alberto Pastor; Responsible person of the Project: Salvador Martinez).

**FIGURE 1:**
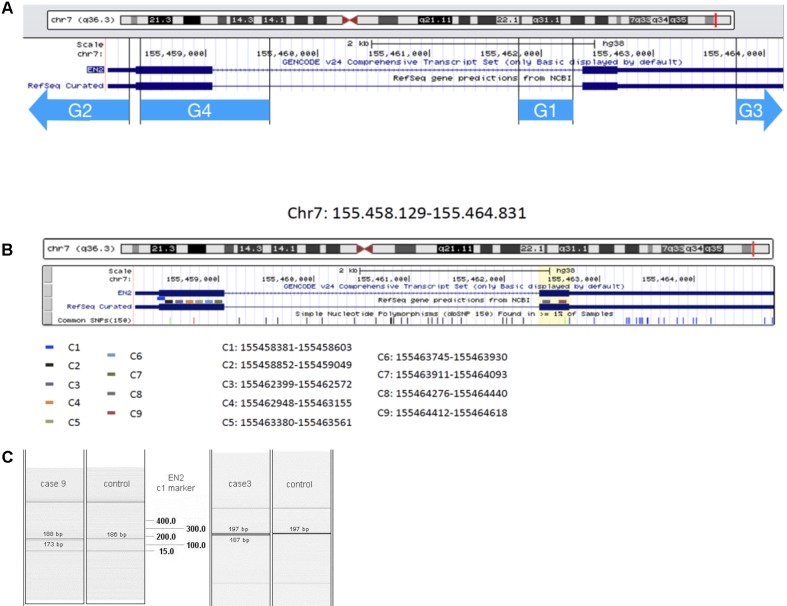
EN2 genomic transcriptomic map. The region analyzed by the genomic markers G1–G4 **(A)** and transcriptomic marker-probes C1–C9 **(B)** is represented by different color lines. **(C)** QIAxcel system data showing results from transcriptomic analysis of C1 marker: in cases 9 and 3, double bands appear at the level of EN2 normal transcription of this region of Exon 1.

*EN2* gene is located on chromosome 7q36 (Chromosome 7, NC_000007.14 (155458129–155464831^1^). *EN2* RNAm is detectable in human peripheral blood: available information in genecards.org by RNAseq and Microarray, Hnoonual et al. (2016) and the present study. Searching for genomic and transcriptomic alterations in this gene we analyzed DNA and cDNA sequences, respectively, by PCR technique. We have studied four DNA markers (G1, G2, C3, and G4, see **Table 1** and **Figure 1A**), and nine complementary DNA markers (cDNA), in its coding region (**Table 2** and **Figure 1B**). Primers for cDNA analysis were designed with Primer-BLAST^2^ and Primer3′; version 0.4.0^3^.

**Table 1:** DNA markers.

DNA MARKER	LOCALITATION ON CHROMOSOME 7q36	PRIMERS SEQUENCE	SIZE
G1	155462376–155462491 bp	G1-F: AGGTCTCGAAAACCAAAGAAG1-R: AGGTACCTGTTGGTCTGGAA	116 bp
SHGC-172649_G2 (no designed by authors)	155456462–155456780 bp	G2-F: TAAGACTTCAAAACCAAGTCGCCG2-R: TTGGTGGGTAGACAAGAGCAAAT	319 bp
G3	155464018–155464926 bp	G3-F: ACCAGGCGTGTTTGAGTCG3-R: GGCCATGAGCACCTGAGT	909 bp
G4	155458129–155459064 bp	G4-F: TCTCTCATCGTCTGGGCGAGG4-R: GCATTGTTTAGCGCGGACTG	936 bp

*From http://www.ncbi.nlm.nih.gov/gene/2020, markers.*

*http://genome.ucsc.edu/cgi-bin/hgPcr?hgsid=609684321_GSEVOB7Lh1Idbn3pCw7akIRTgZIi.*

**Table 2:** cDNA markers.

cDNA MARKER	LOCALIZATION ON EN2 COMPLEMENTARY SEQUENCE (NM_001427)	PRIMERS SEQUENCE	SIZE
C1	253–475 bp	C1-F: GAGGAGAATGACCCCAAGCC1-R: GCAGGATGTTGTCGATGAAG	223 bp
C2	724–921 bp	C2-F: AAGACGCTCTCGCTGCACC2-R: GTCCGAGTAGCGCGTACAGT	198 bp
C3	963–1136 bp	C3-F: GAACCCGAACAAAGAGGACAC3-R: CGCTTGTTCTGGAACCAAAT	174 bp
C4	1493–1719 bp	C4-F: GGCTGCTTAGGGTTTCACCTC4-R: CACCAAGCCAACACAAACAA	208 bp
C5	1944–2125 bp	C5-F: CACCCTCCTGCACCTAACTCC5-R: GACGCAGACGATGTATGCAC	182 bp
C6	2309–2494 bp	C6-F: GTGACTCCACCAGCCATCATC6-R: AAGCAGCCACTCCAAGAAAA	186 bp
C7	2475–2657 bp	C7-F: TTTTCTTGGAGTGGCTGCTTC7-R: TCCTGGAGGATTCTGAGTTCT	183 bp
C8	2840–3004 bp	C8-F: CGCCACACTGTCTTCTGTTTC8-R: AAGAGCGAAGTTCACCCTCA	165 bp
C9	2976–3182 bp	C9-F: CCTGAGGACTGAGGGTGAACC9-R: AACCAACTTTGCTTCCTGCT	207 bp

*Sequence ID:NM_001427.3.*

We extracted DNA and RNA from whole blood using QIAamp DNA Mini Kit (catalog number 51306, Qiagen, Madrid) and RNeasy Mini Kit, respectively (catalog number 74106, Qiagen, Madrid) with a robotic workstation for automated purification of DNA and RNA (Qiacube, catalog number 9001292, Qiagen, Madrid). The cDNA synthesis was made with High Capacity cDNA Reverse Transcription Kit (Part Number 4368814, Applied Biosystems Life Technologies). Concentration and purity were checked with NanoDrop 1000 Spectrophotometer (SID-10135606, Thermo Scientific). We used samples with 30 ng/μl normalized concentration and ratio (A260/A280) between 1.4 to 1.9 values.

The molecular analysis was carried out by multiplex (QIAGEN Multiplex PCR Kit, catalog number 206145) using the primers described in **Table 1**, and the results visualized with QIAxcel System (capillary electrophoresis device, catalog number 9001421, Qiagen, Madrid).

The results of the genetic study distributed the patients in three groups: 12 patients presented mutations in *EN2* gene (EN2-g), *n* = 12, 20 patients presented mutations in other genes studied in the panel used in our laboratory facilities to detect genetic anomalies in patients with diagnostic of cortical dysplasia (*LIS1[PAFAH1B1], PTAFR, PAFAH1B2, PAFAH1B3, FGF8, PAX2, D17S79, D17S1866*, and *D17S5*, **Table 3**), but not in *EN2*, (OG-g); and 62 patients showing neither alterations in genes of the panel or *EN2* (**Table 4**). The statistical association of the clinical characteristics among the three groups was compared by means of association statistical test (X^2^ with a confidence interval 95%).

**Table 3:** Other genes markers.

MARKER	LOCALIZATION	PRIMERS SEQUENCE	SIZE
LIS1 (PAFAH1B1)	729–1232 NM_000430.3	LIS1-F: AATGGATTCCCCGTCCGCCA LIS1-R: CTACACGCACAGTCTGGTCATTGG	504 bp
HLIS1 coding region (PAFAH1B1)	4240–4562 NM_000430.3	HLIS1-F: GCCTGGGATAAGGACAATGA HLIS1-R: ATATTTGGGTGGCACTGGAA	206 bp
HLIS3 coding region (PAFAH1B1)	911–1656//268–1013 NM_000430.3	HLIS3-F: CAGGACATTTCATTCGACCA HLIS3-R: TGATCTACGCTGCCAGTGAC	746 bp
HLIS4 coding region (PAFAH1B1)	2681862–2682065 NC_018928.2	HLIS4-F: GGTTACCCCATTGAGCTCTG HLIS4-R: TTCTTAGGCCTGGTGTGACC	204 bp
HLIS5 coding region (PAFAH1B1)	2732–2933 NM_000430.3	HLIS5-F: GGTTGGAGCGTGCATAAAAATGT HLIS5-R: CTAAAGCATGGCATTCCACA	202 bp
D17S379 locus in 17p13.3	2522387–2522545 NC_000017.11	F-PRMER: GTTGGAACAGAACTATGAATAAC R-PRMER: CCTAACTGAATGACATGGAGGAC	159 bp
D17S1866 locus in 17p13.3	232780–23295	F-PRMER: TGGATTCTGTAGTCCCAGG R-PRMER: GGTTCAAAGACAACTCCCC	167 bp
D17S5 locus in 17p13.3	2096690–2096882 NG_033980.1	F-PRMER: GCCTACCTTCCACAAATCTTTC R-PRMER: TTGCTGGAGGGATACCTGTGTAC	193 bp
PAFAH1B2 (ALFA1)	3305–3483 NM_001309431.1	ALFA1-F: GATTAAGGGGCCAACTTTCC ALFA1-R: ACCCGTGGCAGTACATCTTC	179 bp
PAFAH1B3 (ALFA2)	294–467 NM_001145939	ALFA2-F: ACTTTGGCATTGGTGGTGAC ALFA2-R: GCTCATTCACCAGTTGCACA	174 bp
PTAFR	28149985–28150517	PTAFR-F: AGCAGGGACTAATTTTTGAGG PTAFR-R: AACGTCACTCGCTGCTTTG	533 bp
FGF8	101770456–101770591 NM_001206389.1	FGF8-F: TCACGGAGATTGTGCTG FGF8-R: AAGTGGACCTCACGCTG	136 bp
PAX6	31802704–31802834 NM_001310161.1	PAX6.1-F: GTCACAGCGGAGTGAATCA PAX6.1-R: CTGCAGAATTCGGGAAATGT	131 bp

**Table 4:** Frequencies of the different clinical conditions depending on the genetic results groups.

Group	MR	EP	BD	CP	“n”
EN2-g	12	9(MR + EP)	8(MR + BD)	4(MR + CP)	12
	5(MR + EP + BD)				
	6(MR + EP + BD + CP)				
OG-g	20	14	12	7	20
EN2-g vs. OG-g “*p*” value	ns	ns	ns	ns	
NM-g	40	15	7	7	62
EN2-g vs. NM-g “*p*” value	0.013	0.001	0.0001	0.07^∗^	

*X^2^ test applied. ^∗^Non-significant value after fisher correction for two ties was applied because less than five cases appeared in one cell.*

## Results

### Patients’ Genotype and Phenotype Description

Patients’ genotype: fifteen out of 109 patients were not included in the present study because showed one or more of the exclusion criteria. From the remaining 94 patients, 12 (12.8%) show mutations in *EN2* gene (EN2-g), and five of them showed simultaneously other alterations in the genetic panel applied (**Table 4**). Twenty patients (21.27%) showed mutations in the panel of genes different from *EN2* (OG-g); and finally, 62 (65.96%) have not showed anomalies in this panel (Non-mutations group, NM-g) (**Table 4**). EN2-g patients showed *EN2* genetic deletions in genomic DNA and/or cDNA (**Table 5**), that did not correspond to reported alteration in *EN2* expression splicing, single-nucleotide variants or polymorphisms.

**Table 5:** Resume of the clinical, genetic and MRI characteristics of the EN2-g.

	Gender	EN2 mutation chromosome 7q36	Other mutations (markers)	CP	MR	Autism	Epilepsy	MRI
Case 1	M	Del. 909 bases (bases 155464018–155464926); G3 marker		(+)	Mild	PD	Gen	Asymmetric CSC atrophy
Case 2	F	Del. 207 bases (bases 2976–3182); C9 marker	LIS1, HLIS1		Severe	No	Gen	Hyper intense T2 signal, semiovale center CC atrophy Blake cyst.
Case 3	M	Del. 182 bases (bases 253–475); C1 marker Del. 208 bases (bases 1493–1719); C4 marker	HLIS3	(+)	Severe	No	Partial	CSC atrophy; CC atrophy
Case 4	M	Del. 116 bases (bases 155462376–155462491); G1 marker Del. 208 bases (bases 1493–1719); C4 marker	-		Moderate	-	Gen	CSC atrophy; CC atrophy
Case 5	M	Del. 116 bases (bases 155462376–155462491); G1 marker	-		Mild	Nuclear	Infantile spasm	Hyper intense T2 signal, semiovale center Macrocephaly
Case 6	M	Del. 116 bases (bases 55462376–155462491); G1 marker	LIS1, HLIS1, HLIS5	(+)	Severe	-	2ry Gen	Asymmetric CSC atrophy Left temporal arachnoids’ cyst
Case 7	M	Del. 909 bases (bases 155464018–155464926); G3 marker			Mild	Mild	2ry Gen	Asymmetric CSC atrophy Communic. hydrocephalus Macrocephaly
Case 8	M	Del. 182 pb in C1 marker sequence (bases 253–475)			Mild	Mild	2ry Gen	Hyper intense T2 signal, semiovale center. Microcephalus
Case 9	F	Del. 182 pb in C1 marker sequence (bases 2475–2657)	D17S5	(+)	Severe	No	No	Microcephalus
Case 10	F	Del. 182 bases (bases 2475–2657), C7 marker			Mild	PD	Gen	Microcephalus Hypomielinization of the corpus callosum and corticoespinal tracks
Case 11	F	Del. 182 bases (bases 1944–2125), C5 marker			Mild	PD	No	Hyper intense T2 signal, semiovale center
Case 12	M	Del. 183 bases (bases 2475–2657), C7 marker			Mild	PD	FCs, Gen	Hyper intense T2 signal, semiovale center

*M, male; F, female; Del., deletion; MR, mental retardation; Gen, generalized epilepsy; PD, pervasive disorder; CSC, cortico-subcortical; CC, corpus callosum; MRI, magnetic resonance image.*

Clinical phenotype: the entire EN2-g patients group suffered MR from moderate to severe; nine suffered from EP, eight from BD and four from CP (**Table 4**). Five patients showed a comorbid combination of MR, EP and BD. Four patients showed a comorbid combination of MR, EP, BD, and CP. Therefore, the comorbid presentation of the clinical picture on EN2-g was in the 75% of the cases (9/12; **Table 4**).

Three out of the 12 *EN2* patients came from Maghrebi background, while only one out of the 20 with OG-g belonged to this ethnicity, which does not represent significant differences. However, when we compared with NM-g where no patient belongs to this ethnic background, statistical differences become significant (X^2^ = 19.41; *p* = 0.002).

In relation to observed MRI structural anomalies in EN2-g patients: one patient showed Blake cyst (Case 2); five patients (Cases 1, 3, 4, 6, and 7) showed cortico-subcortical (CSC) atrophy; Cases 2 and 3 presents also corpus callosum atrophy; five cases (Cases 2, 5, 8, 11, and 12) showed hyperintense white matter signal; Cases 9 and 10 showed microcephaly and Cases 5 and 7 showed macrocephaly (**Table 5**). We did not record abnormalities or differences in cerebellar structure among groups, except Blake cyst in IV ventricle and cisterna magna of Case 2. Other minor MRI findings were equally distributed between groups.

The frequency of the different clinical pictures in the OG-g and NM-g are shown in **Table 4**). While MR was present in 100% of patients with genetic anomalies (EN-g and OG-g), only 64.5% patients without present panel of gene alteration showed MR. In addition, while the 75% of EN2-g and 70% of OG-g showed epilepsy, only the 37% of NM-g showed this clinical condition. The percentage of comorbidity in OG-g was 14 out of 20 (70%) with no statistical differences when was compared with the comorbidity of EN2-g. Finally, in NM-g 23 out of 62 (37.1%) showed no comorbidity, and when was compared with EN2-g the comorbidity was significantly lower (X^2^ = 4.43; *p* = 0.04)

The detailed clinical picture of the 12 patients was as follow:

Case 1: A Caucasian 9.4-years-old boy followed because of a severe neurodevelopmental delay and epilepsy. Pre- and perinatal history was normal. At the age of three months old he showed irritability and gastroesophageal reflux. A severe neurodevelopmental delay rose at the age of four months. The patient showed a severe partial refractory epilepsy and severe tetraplegic cerebral palsy. He did not develop any form of language, eye-to-eye contact, neither basic motor nor social milestones. Cytogenetic, metabolic panels, including studies for mucopolysaccharidosis, organic acids, amino acids, long chain polyunsaturated fatty acids, and muscle biopsy were normal. The EEG records showed immature rhythms for the age of the patient. No paroxysmal events were recorded in the last two years before enrolling follow-up. The serial MRI studies showed a static cortico-subcortical atrophic pattern with marked asymmetry.

The genetic study showed 909 bases deletion in *EN2* genomic sequence, G3 marker (155464018–155464926 bases).

Genetic analysis of *LIS*, *PTAFR, PAFAH1B2, PAFAH1B3, FGF8*, and *PAX2* genes did not reveal alterations.

Case 2: A Maghrabian14-month-old girl, followed because of neurodevelopmental delay and epilepsy. The patient has antecedents of gestational diabetes and delivery by cesarean section at the gestational age of 40 weeks. She also showed developmental delay and infantile spasms. The high definition karyotype and the basic metabolic panel were normal. The EEG record showed slow waves, sharp waves, and spikes and waves discharges. The MRI showed a Blake cyst in posterior fossa, corpus callosum atrophy and hyperintense signal in the white matter of the semiovale centrum, which was related to the normal myelinisation process. The combination of valproic acid and vigabatrin induced the remission of the seizures and a mild neurodevelopmental delay was persistent.

The genetic study showed 207 bases deletion, due to transcription problems in the *EN2* exon 2 (C9 marker) (bases 2976–3182) (C#9 in **Figure 1**).

The patient also showed 504 bases deletion in LIS1 marker region of *PAFAH1B1* (from 729 to 1232 bases) and a 206 bases deletion in HLIS1 marker of *LIS1[PAFAH1B1]* coding region 4240–4562 (from 4357 to 4562 bases). No alterations were found in genes *PTAFR, PAFAH1B2, PAFAH1B3, FGF8*, and *PAX2*.

Case 3: A Caucasian 6.5 year-old boy was followed by neonatal hypotonia without pre and perinatal significant antecedents. The patient showed a severe developmental delay, severe mental retardation with choreoathetosis, tetraparesis, cerebral palsy, and reflex generalized epilepsy. The physic exam showed microcephaly, weight, and stature delay. The patient did not develop language, gait, or even steady sitting. He also showed incessant choreoathetosis, which sometimes reminded stereotypies, and eye-to-eye contact making some social liaison with close relatives and immediate people surrounding him. The EEG showed abnormal discharges of slow waves and spikes and waves. The background rhythm was slow and immature for the age. The MRI showed cortico-subcortical generalized atrophy and corpus callosum atrophy. The treatment with valproic acid improved the reflex epilepsy. He also needs intensive treatment with physiotherapy. Head control is the unique milestone acquired at the moment.

The genetic study showed transcription problems, 208 bases deletion in C4 marker (1493–1719 bases) and 182 bases deletion, in C1 marker (bases 253–475) of the *EN2* gene (C#4 and C#1 in **Figures 1B,C**). The patient also showed a 746 bases deletion in the HLIS3 marker (codifying sequence: 911–1656, 268–1013) of the *LIS1* gene. No alterations were found in genes *PTAFR, PAFAH1B2, PAFAH1B3, FGF8*, and *PAX2*.

Case 4: A Maghrebian 8.2 year-old boy. Coming from difficult social background, so no perinatal antecedents were available. The patient was referred for evaluation because of mild mental retardation and hyperactive behavior. He also showed history of partial secondary generalized epilepsy with persistent atypical absences and psychogenic episodes. The physic and neurological exam were normal. The patient showed moderate mental retardation with language delay. The EEG showed abnormal discharges of slow waves and spikes and bi-temporally localized waves. The MRI study showed cortico-subcortical atrophy and corpus callosum hypoplasia. A high definition karyotype and metabolic panel were normal. The seizures and the hyperkinetic behavior improved after the administration of valproic acid.

The genetic study showed 116 bases deletion in genomic sequence of *EN2* gene: 155462376–155462491 (G1 marker). The patient showed abnormalities in the transcription of the *EN2* gene, that implies the deletion of 208 bases (between 1493 and 1719 bases of the complementary sequence, C4 marker). (C#4 in **Figure 1**).

In *LIS1, PTAFR, PAFAH1B2, PAFAH1B3, FGF8*, and *PAX2* genes no alterations were found.

Case 5: A mixed Maghrebian/Caucasian ethnicity 12 years-old girl, without pre and perinatal antecedents, followed in the outpatient’s clinic because of severe mental retardation, nuclear autistic condition, macrocephaly, severe language delay, neurosensorial hypoacusia, and joint malformations with elbows internal rotation, hip dysplasia that conditioned duck gait, and tarsal malformation, which conditioned flat plants. Facial features were peculiar with flat philtrum, wide nasal wings, bilateral epicanthus, prominent forehead, and low implantation ears. The EEG showed a delayed rhythm pattern. High definition karyotype and metabolic panel as well as organic acids, blood and urine amino acids, mucopolysaccharides in urine, medium, and long chain fatty acids, and a TORCH study were normal. MRI showed hyperintense signals in both posterior and anterior semiovale centrum. These leukodystrophy lesions did not changed throughout the seven years MRI follow-up. As the patient breaks into adolescence, the autistic behavior worsened with self-aggressions and stereotypies, which slightly improved with a risperidone and carbamazepine combination.

The genetic study showed 116 bases deletion in genomic sequence of *EN2* gene: 155462376–155462491 (G1 marker).

Genetic analysis of *LIS1*, *PTAFR, PAFAH1B2, PAFAH1B3, FGF8*, and *PAX2* genes no showed alteration.

Case 6: A Caucasian 2.2 year-old boy without pre and perinatal antecedents was admitted to the outpatients’ clinic because a specific learning disorder. Neurological history showed developmental delay and hypotonia, with clearly retarded motor and cognitive milestones. The somatometry values were normal. Maturational EEG was normal. MRI showed left temporal arachnoids’ cyst and cortico-subcortical asymmetric atrophy. General metabolic panel and high definition karyotype were normal. The physic and neurological exam were also normal. With the diagnosis of specific learning disorder the patient was included into a psycho pedagogical teaching program.

The genetic study showed 116 bases deletion in genomic sequence of *EN2* gene: 155462376–155462491 (G1 marker).

The patient also showed a transcriptional error in LIS1: a 504 bases deletion in LIS1 marker region (from 729 to 1232 bases; 206 bases deletion in HLIS1 marker *LIS1[PAFAH1B1]* coding region (4240–4562) (from 4357 to 4562 bases) and a 202 bases deletion in HLIS5 marker of *LIS1[PAFAH1B1]* coding region (2732–2933). Genetic analysis of *PTAFR, PAFAH1B2, PAFAH1B3, FGF8*, and *PAX2* genes no showed alteration.

Case 7: A10-month-oldCaucasian boy without perinatal antecedents attends to the outpatient clinic because of macrocephaly (49.5 cm + 2SD) and mild developmental delay. Ultrasound scan and MRI studies confirm left hemispheric asymmetric cortico-subcortical atrophy and benign communicating hydrocephalus. Follow-up sustained the developmental delay and episodic abnormal gait with right hip claudication. Homogenous spleen growth was detected at the age of two. Infectious and metabolic panels did not reveal any abnormalities. The patient follow-up remains still open, but no changes have been obtained on the MRI and metabolic studies.

The genetic study showed 909 bases deletion, due to transcription problems in the *EN2* (G3 marker) (genomic sequence: 155464018–155464926 bases). Genetic analysis of *LIS1*, *PTAFR, PAFAH1B2, PAFAH1B3, FGF8*, and *PAX2* genes no showed alteration.

Case 8: A 12-year-old Caucasian boy referred to us to be evaluated because of moderate mental retardation, microcephalus and gait impairment. Perinatal deleterious events were not mentioned in the patient historic reports. Developmental delay has been a constant feature during his follow-up as well as microcephalus. Over the age of fifteen a complex obsessive-compulsive behavior arose. On the other hand, MRI, apart for microcephaly, as well as metabolic and infectious tests was normal. A slight ataxic gait with little knee flexion has been recorded during the follow-up.

The patient was heterozygous for the EN2 markers showing anomalies in the transcription-codifying region, which cause 182 bases deletion in one of the alleles (between 2475 and 2657 bases of the complementary sequence, C7 marker). (C#7 in **Figure 1**).

On the other hand, analysis of *LIS1*, *PTAFR, PAFAH1B2, PAFAH1B3, FGF8*, and *PAX2* genes no showed alterations.

Case 9: A 16-year-old Caucasian female in follow-up in the outpatient’s clinic with the diagnosis of cerebral palsy of unknown origin although complete metabolic and genetic workouts have been done. A severe MR and CP with slightly progressive motor dysfunction were registered. The family reported no epileptic episodes. The MRI studies showed a normally structured encephalon but microcephalus.

The patient was heterozygous for the EN2 markers showing anomalies in the transcription-codifying region, which cause deletion of 182 bases in one of the alleles (between 253 and 475 bases of the complementary sequence, C1 marker). (C#1 in **Figures 1B,C**).

She also showed heterozygous abnormalities in the D17S5 marker that is localized in the lissencephaly critical region (17p13.3), causing a four bases deletion (between 2096690 and 2096882 bases).

Genetic analysis of *PTAFR, PAFAH1B2, PAFAH1B3, FGF8*, and *PAX2* genes no showed alteration.

Case 10: A 3.5 year-old Caucasian girl was referred to us because of gait impairment and microcephalus. At the age of five, she presented an episode of disconnection of the environment during few seconds. The EEG records were normal at the moment of the episode, although records of isolated febrile convulsions and disruptive sleep disorders have been obtained. She recovered from the gate problems, but during the pre-school year she showed a developmental delay mainly motor. At the beginning of the school time, a notorious behavior problem arose resembling an ADHD that requires psycho-pedagogic intervention and treatment with methylphenidate, obtaining discrete results at school performance but a worsening in the sleeping problems, so a new EEG is pendant. The MRI showed a normally structured encephalon but also with microcephalus.

The genetic study showed 182 bases deletion, due to transcription problems in C7 marker (bases 2475–2657). (C#7 **Figure 1**).

No alterations were found in genes *LIS1*, *PTAFR, PAFAH1B2, PAFAH1B3, FGF8*, and *PAX2*.

Case 11: A 6 year-old girl of Romany ethnical background was followed because developmental delay, poor school performance and gait impairments. The MRI showed a hyperintense signal, especially in the right semi-oval center, which has been steady during successive MRI studies. The gait impairment has solved, but certain degree of motor clumsiness remains. The learning problems worsened and now she needs curricular adaptation. The school reports suggest a borderline intelligence or a mild mental retardation.

The patient showed abnormalities in the transcription of the *EN2* gene, that implies the deletion of 182 bases (between 1944 and 2125 of the complementary sequence, C5 marker). (C#5 in **Figure 1**).

No alterations were found in genes *LIS1*, *PTAFR, PAFAH1B2, PAFAH1B3, FGF8*, and *PAX2*.

Case 12: A 2.3 year-old boy of South American ethnical background, under a public social institutions care, was referred to us presenting microcephalus, complex febrile seizures, and moderate developmental delay. There were records of prenatal exposure to ethanol. The MRI study showed hyperintense lesions in both “corona radiata” more evident in the right side. The spectroscopy study suggests lack of maturity of the frontal lobe, parietal areas, and basal nuclei. EEG studies reflect the presence of abnormal activity with generalized slow pattern and theta and delta bursts bilaterally in temporal regions, both in sleep and awake states. He was treated with valproic acid with an adequate response both physiologic and clinic.

This patient shows abnormalities in the transcription of *EN2* gene with the deletion of 183 bases (2475–2657 bases, C7 marker). (C#7 in **Figure 1**).

No alterations were found in genes *LIS1*, *PTAFR, PAFAH1B2, PAFAH1B3, FGF8*, and *PAX2*.

## Discussion

**FIGURE 2:**
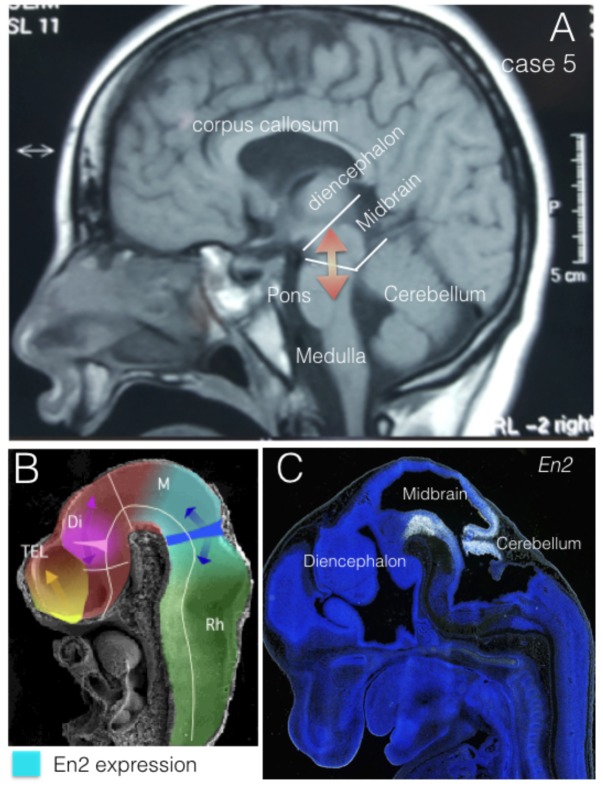
EN2 expression regions. **(A)** Midsagittal NMR section from case 5. Midbrain and cerebellar regions have been identified as Mid-Hindbrain regions where EN2 expression plays its morphogenetic role (red arrows). **(B)** Midsagittal section of an E10 mouse embryo in scanning electron microscopy, where it has been represented the expression domain of *En2* (light blue) in relation to the expression of *Fgf8* (dark blue) and the morphogenetic activity of the isthmic organizer (dark blue arrows). **(C)** EN2 expression by in situ-hybridization at the Mid-Hindbrain region (caudal midbrain and cerebellum) in a mouse embryo at E13.5. From The Allen Institute, Developing Mouse project, data-base (http://developingmouse.brain-map.org/).

The engrailed homeobox protein (EN) distribution in the mid-hindbrain segment is fundamental to understand the development and function of the isthmic organizer that regulates the cerebellum and midbrain morphogenesis (**Figure 2**). At neural tube stages, the isthmic organizer signal, Fgf8, determine the central band of high level of EN expression at the isthmus, and the bilateral decreasing expression of EN over mesencephalic and rhombencephalic neuroepithelium (Crossley et al., 1996; Martinez, 2001). This gradiental distribution of EN protein has been demonstrated as a mechanism of positional information required for topographic organization of synapsis in the retinotectal system (Nakamura and Sugiyama, 2004). In addition, EN expression is required to specify serotoninergic and dopaminergic neurons in the hindbrain, which control cerebral cortex function trough raphe-cortical and meso-cortical projections, respectively. Therefore, alterations of EN expression might be associated to synaptic anomalies underlying neurodevelopmental disease in humans. Actually, genetic alterations in the expression of *EN2* have been related to different neurologic conditions and more particularly to autism spectrum disorder (ASD).

ASD is a neurodevelopmental disorder with a strong genetic component. A close relationship between ASD and *EN2* specific polymorphisms, as well as genetic and epigenetic alterations, has been established in several studies, suggesting that the etiologic influence of *EN2* variants in ASD may be explained through genetic mechanisms (Petit et al., 1995; Blair et al., 2002; Brune et al., 2008; Benayed et al., 2009; Sen et al., 2010; Viaggi et al., 2015). Although the underlying genetic architecture is not well understood, two theories explain the genetic roots of common complex diseases, including the ASD. The first is the common variant-common disease hypothesis (CVCD), which suggest that ASD is due to a combination of frequent genetic allelic variations (>1%), each conferring a modest risk (odds ration < 1.5) (Bodmer and Bonilla, 2008). The second one is the rare variant-common disease (RVCD) hypothesis, in which the genetic risk of ASD is explained by rare mutations conferring a significant risk (Talkowski et al., 2014). Since the RVCD is favored by recent studies on genetic causes of ASD (Huguet and Bourgeron, 2016; Liu and Takumi, 2014), we have studied in the present work the potential contribution of rare gene variants in *EN2* to ASD phenotype. The analysis of *EN2* gene alterations and the presence of ASD phenotypes did not support a significant relation between *EN2* and ASD in our selected population of patients showing encephalic structural anomalies (ESA). We have demonstrated that *EN2* gene alterations contribute to phenotypes of ESA, but they are not more related to ASD than other genetic anomalies. Nevertheless, 8 of 12 EN2-g patients have exclusively mutations in *EN2* and 6 of them showed ASD of different degrees: Cases 1, 10, and 12 showed PD; Case 5 showed nuclear autism, Cases 7 and 8 showed mild autism, and Cases 4 and 2 did not show autism; suggesting a potential contribution of EN2-related developmental processes in autism physiopathology. But, the lacking of massive genomic and transcriptomic studies did not allow us to eliminate a pathogenic influence of other genetic alterations. In addition, we have not studied *EN2* expression in ASD patients without ESA, where pathogenic influence of EN2 protein expression may become evident.

Regarding the possibility that the ethnic difference might confound the *EN2* association with autism, five out of twelve EN2-g patients have three different ethnical backgrounds apart from Caucasians (three Maghrebian, one Romany, and one South American). Although the sample is not large enough to be conclusive, it could be suggestive of ethnic association between *EN2* gene alterations and brain anomalies in groups with higher traditional consanguinity.

The main feature associated to the reported *EN2* abnormalities in this study, is MR, but this was similarly expressed among OG-g patients who did not show these *EN2* abnormalities. However, MR was far less frequent among those patients of NM-g. There are few series studies reporting *EN2* and MR association (Laroche et al., 2008), although it seems to be universally spread among single case reports (Sarnat et al., 2002; Titomanlio et al., 2005). In our study, as mentioned before, all the EN2-g patients suffered from some intellectual disability, ranging from severe to mild MR, in agreement to the described phenotype in 7q terminal deletion syndrome where *EN2* gene is affected (Frints et al., 1998; Linhares et al., 2013). Moreover, axonal guidance in target territories to establish adequate synaptic patterning depend of engrailed proteins concentration (Marie and Blagburn, 2003); then, decreasing of *EN2* transcription could represent a haploinsufficient phenotype that predispose to MR and ASD in 7q terminal deletion syndrome. Moreover, recent findings *En2* knockout mice display reduced levels of tyrosine hydroxylase, noradrenaline, and serotonin in the hippocampus and cerebral cortex, similar to those observed in human brains with ASD (Viaggi et al., 2015). In addition, Soltani et al. (2017), have reported an effect of En2 protein in neuronal morphogenesis and synaptogenesis of hippocampal neurons in culture. Therefore, although other associate genetic anomalies have been detected in EN2-g patients that may explain the ESA phenotypes, *EN2*-related alterations in cortical development and neuronal differentiation might be related to the presence MR in all of EN2-g patients. In addition, similar alterations in *LIS1* related genes have been demonstrated in patients suffering schizophrenia and bipolar disorder (Tabares-Seisdedos et al., 2006), in favor of common pathogenic mechanisms underplaying neurodevelopmental disorders.

In our study, EP was not more frequent among EN2-g patients than it was in the rest of the OG-g series (75 and 70%, respectively). However, it is more frequent than in NM-g (37%). There were scarce literature published about the linkage between the *EN2* mutations and epilepsy, although it may be expected because its theoretical relations with ASD (Tuchman et al., 2010). However, in mice models, Tripathi et al. (2009) found that Hippocampal *En2* mRNA content decreased after seizures induced by kainic acid (KA). This suggests that *En2* might also influence the functioning of forebrain areas during adulthood and in response to seizures (Tripathi et al., 2009; Tripathi and Bozzi, 2015). Moreover, our EN2-g, show in four cases, transcriptional errors in *LIS1*, *HLIS1, HLIS3*, and *HLIS5*, markers of *LIS1[PAFAH1B1]* gene, which are also present in the OG-g patients, and have been related to a wide variety of phenotypic abnormalities associated to EP (Ross, 2002).

Only four of the EN2-g patients showed gross motor impairments resembling CP, which has not been described before. Three out of four, showed also mutations in the critical region of lissencephaly (*LIS1* markers; see **Table 5**) that can explain the macroscopic structural abnormalities associated to migration anomalies and cortical dysplasia, as has been reported in animal models and patients (Wurst et al., 1994; Yang et al., 2008; Cheng et al., 2010; Hadjivassiliou et al., 2010).

Cerebellar malformations were expected more frequent in EN2-g, attending to previous studies (Millen et al., 1994; Wurst et al., 1994; Blair et al., 2002; Spalice et al., 2009; Viaggi et al., 2015), but differences were not detected among our groups. The *En1* and *En2* genes have been proved sharing co-expression domains in the developing cerebellum and mesencephalon of mice (Millen et al., 1994; Cheng et al., 2010; Wilson et al., 2011). Although important anomalies have been related with down regulation of *En1* and *En2* knockouts in mouse mutants (Millen et al., 1994; Wurst et al., 1994; Cheng et al., 2010; Wilson et al., 2011) the possibility of mutual compensatory activity has been also proved (Hanks et al., 1995).The absence of systematic and significant cerebellar anomalies in humans with genetic alterations in *EN2* may be consequence of this compensatory effect in the heterozygous background.

Neurological comorbidity rate was similar among EN2-g and OG-g patients and was less frequent among NM-g patients. Our findings suggest that the dysfunctions of genes evaluated in this study, which regulate the structural development of the CNS, induce a wide range of clinical pictures that involve most of the functional areas of the brain, resulting in a small group of neurological syndromes clinically recognizable. This would suggest that the clinical picture that they show is the product of a suboptimal brain function due to the miss organization of the brain structures. In practice, from a clinical point of view, these patients can be resumed in a group with abnormal adaptive conditions of the directive functions of the CNS, which can be more or less evident depending on the epigenetic and environmental factors and induce the similar suboptimal cognitive patterns, even with heterogeneous genetic backgrounds.

Our basic gene and cDNA EN2 screening is a preliminary study about genomic changes that are producing consequences in the transcriptomic profile. Furthermore, experiments using modern sequentiation approaches (e.g., deep sequencing and RNAseq) and in larger patient’s population will be necessary to confirm and define precisely En2 and other gene alterations in congenit brain structural anomalies.

In conclusion, *EN2* gene variations analyzed in peripheral blood samples, altogether with the other genes analyzed in the present study, showed a high prevalence of unspecific clinical pictures, including ASD, as well as a major mutual comorbidity. We have not detected clear differences in the prevalence of behavioral disturbances, especially ASD between patients showing *EN2* anomalies and patients with other genetic anomalies. This unspecific pattern supports the polygenic nature of ASD comprising the novo and rare inherited variants acting within the context of rare-variant genetic load (revised in Chaste et al., 2017).

## Ethics Statement

Clinical data are related to a descriptive observational study on 12 patients showing EN2 mutations (Group: EN2-g), out of 109 patients coming from a referral pediatric population (under fifteen years old) of 33.291 patients, was carried out with the approval of the ethics. The reference code ethical approval to use human samples (including blood cells) is: 2016/VSC/PEA/00091 (Responsible person of Ethical Committee: Alberto Pastor; Responsible person of the Project: Salvador Martinez).

## Author Contributions

FC-M conceived and designed the work, analyzed and interpreted the clinical data, drafted the work, revised the manuscript, and agreed to be accountable for all aspects of the work, ensuring its scientific accuracy and integrity. PA-L analyzed and interpreted the clinical data. TE-M and AB-L generated and interpreted the genetic data. MM-M generated and interpreted the genetic and cellular data, and revised the draft. CB designed, generated, and interpreted the cellular and genetic data, revised the draft. SM conceived and designed the work, interpreted the genetic data, revised the draft. PA-L, TE-M, AB-L, MM-M, CB, and SM agreed to be accountable for all aspects of the work, ensuring the accuracy and integrity of the data. All authors approved the final version of the manuscript to be published.

## Conflict of Interest Statement

The authors declare that the research was conducted in the absence of any commercial or financial relationships that could be construed as a potential conflict of interest.

## Footnotes

1 www.ncbi.nlm.nih.gov/gene

2 http://www.ncbi.nlm.nih.gov/tools/primer-blast/

3 http://frodo.wi.mit.edu/primer3/
